# Differentiating gastrointestinal tuberculosis and Crohn's disease- a comprehensive review

**DOI:** 10.1186/s12876-023-02887-0

**Published:** 2023-07-19

**Authors:** Arup Choudhury, Jasdeep Dhillon, Aravind Sekar, Pankaj Gupta, Harjeet Singh, Vishal Sharma

**Affiliations:** 1 Nagaon Medical College Hospital, Assam, India; 2 Ghent University, Ghent, Belgium; 3 Postgraduate Institute of Medical Education and Research, Chandigarh, 160012 India

**Keywords:** Abdominal tuberculosis, Inflammatory bowel disease, Xpert Mtb/Rif, Colonoscopy, Computed tomography, Histopathology, Intestinal tuberculosis

## Abstract

Gastrointestinal Tuberculosis (GITB) and Crohn’s disease (CD) are both chronic granulomatous diseases with a predilection to involve primarily the terminal ileum. GITB is often considered a disease of the developing world, while CD and inflammatory bowel disease are considered a disease of the developed world. But in recent times, the epidemiology of both diseases has changed. Differentiating GITB from CD is of immense clinical importance as the management of both diseases differs. While GITB needs anti-tubercular therapy (ATT), CD needs immunosuppressive therapy. Misdiagnosis or a delay in diagnosis can lead to catastrophic consequences. Most of the clinical features, endoscopic findings, and imaging features are not pathognomonic for either of these two conditions. The definitive diagnosis of GITB can be clinched only in a fraction of cases with microbiological positivity (acid-fast bacilli, mycobacterial culture, or PCR-based tests). In most cases, the diagnosis is often based on consistent clinical, endoscopic, imaging, and histological findings. Similarly, no single finding can conclusively diagnose CD. Multiparametric-based predictive models incorporating clinical, endoscopy findings, histology, radiology, and serology have been used to differentiate GITB from CD with varied results. However, it is limited by the lack of validation studies for most such models. Many patients, especially in TB endemic regions, are initiated on a trial of ATT to see for an objective response to therapy. Early mucosal response assessed at two months is an objective marker of response to ATT. Prolonged ATT in CD is recognized to have a fibrotic effect. Therefore, early discrimination may be vital in preventing the delay in the diagnosis of CD and avoiding a complicated course.

## Introduction

Distinguishing gastrointestinal tuberculosis (GITB) from Crohn’s disease (CD) is a significant clinical problem due to the similarities in the two conditions. GITB and CD are both chronic granulomatous diseases with overlapping symptomatology, radiological, endoscopic, and histopathological findings [1–5]. CD and GITB have different pathogenesis and therapeutic approaches, while they share similarities in their presentation, which makes clinical diagnosis difficult [5, 6].

Crohn’s disease is a type of inflammatory bowel disease (IBD) with a chronic inflammatory process that could potentially involve any part of the gastrointestinal tract and is characterized by skip lesions. CD's pathogenesis results from the complex interactions between the genetic susceptibility of the host and environmental triggers, leading to gut dysbiosis and dysregulated immune response. Genome-wide association studies have identified multiple alleles that are specific to CD. Most genes identified are related to bacterial sensing and innate immunity, like NOD2, LRRK2, IRGM, JAK2, and ATG16L1 [7, 8]. The purported environmental risk factors are cigarette smoking, childhood exposure to antibiotics, drugs like oral contraceptive pills, NSAIDs, and a diet rich in saturated fat and low in dietary fibre [9–11]. The gut microbiota of a patient with CD has a reduction in Firmicutes and Bacteroides, which are in abundance in the healthy population. In a genetically susceptible host exposed to environmental modifiers, the dysbiotic microflora induces mucosal injury to the intestinal epithelium, and the lamina propria T lymphocytes initiate a pro-inflammatory response. The presentation of CD is usually insidious in onset, and clinical features depend on the location of the disease and the behaviour (inflammatory, stricturing, or penetrating) of the disease. The most common clinical presentations are abdominal pain, chronic diarrhea, and weight loss [12]. For patients with colonic involvement, bleeding per rectum may also be seen. Perianal disease is common and seen in around one-third of patients [13].

Tuberculosis is an infectious disease usually caused by *Mycobacterium tuberculosis*which typically involves the lungs but could involve any other organ of the human body [6]. Although considered a disease of the developing world, the developed world continues to see cases because of migration, and in immunosuppressed individuals e.g. human immunodeficiency virus (HIV) related and therapy-related (anti-TNF, steroids) immunosuppression [3]. A majority of cases of tuberculosis in TB-endemic regions may have no identifiable predisposing factors. GITB results in chronic intestinal inflammation, which, like CD, is associated with granuloma formation [6]. The frequent clinical manifestation of GITB is abdominal pain (30–88%), fever (21–73%), diarrhea (5–47%), loss of appetite (30–90%), loss of weight (8–80%) and bleeding per rectum (5–15%) [14]. Patients may also present with obstructive symptoms, right iliac fossa pain, or a palpable mass in the right iliac fossa. GITB can either be of primary intestinal origin or result from the dissemination of pulmonary disease. The most commonly affected sites of GITB are the terminal ileum and ileocecal junction, followed by other regions of the colon and jejunum. Like CD, GITB can involve any part of the gastrointestinal tract (GIT) [3, 6].

### Problem statement and consequences of misdiagnosis

IBD is often seen as a disease of the developed world. However, recent times have seen an increase in the number of patients with IBD, including CD in developing countries, including China and India [15]. These regions are recognized as TB-endemic, and an increase in CD has resulted in a renewed focus on appropriate strategies to discriminate between GITB and CD. It is expected that the numbers of IBD will continue to rise with the shift from UC to CD, making the clinical discrimination of GITB and CD extremely important [16]. A recent report estimated that the overall disease burden of IBD in India is the second highest in the World after the USA [17]. The recent increase in the incidence of CD in India may be related to greater awareness of IBD and better access to endoscopy and imaging modalities. On the other hand, TB cases continue to occur in developed countries in association with HIV and other causes of immunosuppression. Therefore, clinicians across the globe will continue to face challenges in discriminating against GITB and CD [18]. The present review will summarise the available evidence and strategies for discriminating GITB and CD, and formulate possible areas of future research.

Distinguishing GITB from CD is difficult due to similarities in clinical manifestations and poor sensitivity of microbiological tools for the diagnosis of GITB [5]. A South Korean study shows that around 11% of GITB patients were misdiagnosed as CD, while around 18% of CD patients received the diagnosis of GITB [19]. However, the treatment of both diseases differs. Misdiagnosis and implementing the wrong therapy can be dangerous due to possible complications. Misdiagnosis of CD results in delayed treatment for IBD with a risk of disease progression and stricture formation. There is also a risk of adverse effects, including hepatotoxicity due to antitubercular therapy with potentially fatal consequences [1, 2]. Anti-mycobacterial therapy (anti-tubercular therapy or ATT) has been used in the past in the hope that it will help improve CD. A Cochrane review suggests potential benefits with the use of ATT in CD with a lower relapse rate as compared to those not receiving anti-mycobacterial therapy [20]. Improvement in CD symptoms and a decline in inflammatory markers are well recognized with the use of ATT [21–23]. However, there is a growing recognition of the adverse consequences of administering ATT in CD. In a retrospective study comparing patients with CD receiving ATT to those who did not receive ATT, the administration of ATT predisposed patients to stricture formation and the need for surgery. While this could be a consequence of a diagnostic delay or the ‘fibrotic’ effect of ATT, the study seemed to suggest that the cause is not related to the diagnostic delay [24]. On the contrary, another report from India suggested that the empirical ATT is an important contributor to the diagnostic delay that results in stenotic complications and the need for surgery [25]. A recent report suggested that patients with CD who received prophylactic ATT while on anti-TNF therapy have an increased progression to stricturing or penetrating phenotypes [26]. Together, these studies seem to suggest that the use of ATT in patients with CD is not innocuous, and efforts must be made for early discrimination between these two entities. Even if baseline discrimination is not possible, the ATT should be administered with a close follow-up and early colonoscopy performed to ensure early assessment and discrimination [27–29].

Further, misdiagnosing GITB and treating it as CD with steroids or immunosuppressive therapy can cause the dissemination of tuberculosis [30]. Use of immunosuppressive therapy in GITB has been reported to result in the dissemination of tuberculosis, the need for surgery, and even mortality [31]. Therefore, making an accurate diagnosis at the earliest possible stage is essential [1, 4].

### Clinical presentation

Both CD and GITB can present with intestinal and extraintestinal symptoms, and there is considerable overlap between the clinical presentation of the two entities. Both conditions may be associated with abdominal pain, features of intestinal obstruction, and weight loss. However, certain findings may help to discriminate between the two conditions, although none of these findings are specific. The presentation of CD is more prolonged and indolent, while GITB presents with a shorter duration of symptoms (typically < 6 months) [32]. Constitutional symptoms, especially the evening rise of temperature and fever, support the diagnosis of GITB. Fever is infrequent in CD unless complicated by abscess or infection. Although occasional studies suggest age and gender differences in the two conditions, these are not helpful to a clinician in discriminating between the two [1]. Presence of pulmonary symptoms, especially cough, expectoration, and hemoptysis, may suggest pulmonary involvement, which could occur in a subset of patients with GITB [33]. On the other hand, the presence of extraintestinal manifestations, perianal disease, diarrhea, and hematochezia typically favor a diagnosis of CD. A palpable abdominal lump is uncommon in CD but could occur in GITB because of clumped bowel loops, abdominal cocoon or loculated ascites [34].

A Bayesian meta-analysis by Limsrivilai et al. observed that the disease duration was longer in patients with CD than in patients with GITB. [18] In this systematic review, the intestinal symptoms of CD and GITB were abdominal pain (84.3% vs. 84.3%) chronic diarrhoea (63.1% vs 44.4%), recurrent intestinal obstruction (24% vs. 18.8%), hematochezia (34.4% vs. 14.7%), perianal signs (23.3% vs. 3.3%), and constitutional symptoms like fever (29.4% vs. 49.4%) and night sweats (12.3% vs. 39.4%). The extraintestinal symptoms include arthralgia, arthritis, and ocular and dermatological manifestations [35, 36]. However, these symptoms are neither specific to CD nor GITB.

**Fig. 1:**
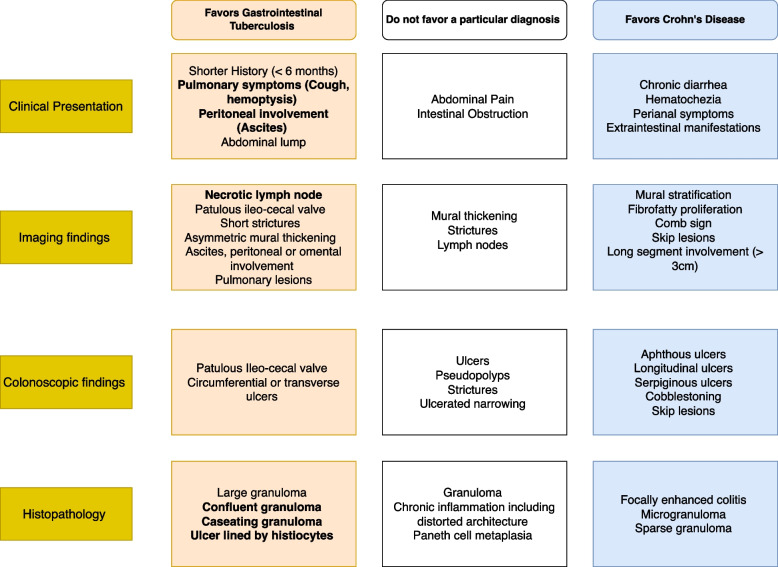
Standard findings on clinical evaluation, serological testing, imaging and histology which could help discriminate gastrointestinal tuberculosis and Crohn’s disease (Bold- specific for the disease)

The pathogenesis of GITB is due to the penetration of the mucosa by the organism, usually after swallowing the infected sputum. Thus the search for active pulmonary infection helps differentiate GITB from CD. However, it should be noted that only 20–25% of GITB patients have concomitant pulmonary involvement [37, 38]. Another clinical feature that helps differentiate GITB from CD is the presence of ascites. Concomitant intestinal and peritoneal involvement can be seen in around 16% of patients with GITB [22]. As tuberculosis can involve the intestine and the peritoneum, exudative ascites favors the diagnosis of GITB over CD [39]. The most common symptoms of GITB are abdominal pain (85%), weight loss (66%), and fever (35–50%). Diarrhoea is observed only in 20% of patients, and in around 25–50% of patients, an abdominal lump is felt in the right lower abdomen [37]. On the contrary, diarrhoea, bleeding per rectum, and perianal diseases are more commonly seen in CD [32, 40]. The clinical features used to distinguish CD and GITB are summarized in Fig. 1.

### Serological evaluation

Tuberculin skin test (Mantoux test) and Interferon-gamma release assays (IGRA) are considered helpful in identifying past TB infection or latent TB. False positive Mantoux reaction can occur due to previous BCG vaccination or could be due to other non-tubercular mycobacterial infections. IGRAs are relatively specific for *Mycobacterium tuberculosis*. The reported pooled sensitivity and specificity of IGRA in differentiating GITB from CD was 74% and 87% respectively [41]. Thus a negative IGRA or Mantoux test does not always help exclude GITB. Further, even patients with CD residing in TB endemic regions could have a positive IGRA due to exposure to TB, further diminishing its benefit in TB endemic regions. A retrospective study from China tried to differentiate GITB from CD based on the cutoff value of TB-IGRA. They reported that TB-IGRA level > 100 pg/ml had a sensitivity of 88% and specificity of 74% for the diagnosis of GITB, and those with TB-IGRA level > 400 pg/ml had a more severe form of GITB [6].

CD is well recognized to have antimicrobial antibodies like Anti-Saccharomyces cerevisiae antibody (ASCA), anti-glycan antibodies, anti-outer-membrane porin C (OmpC) antibody, Anti-flagellin (Cbir1) antibody etc [42]. A meta-analysis found that the pooled sensitivity, specificity, and diagnostic accuracy of ASCA in the diagnosis of CD was 33%, 83%, and 57%, respectively [43]. Another comparative study found that there was no significant difference in the presence of IgA ASCA antibody and IgG ASCA antibody in patients with GITB and CD. They also observed no correlation between ASCA and the disease location, behavior, and disease duration of CD and GITB [44]. In another report, antibodies against zymogen granule glycoprotein GP2 (aGP2) were suggested to outperform ASCA in discriminating GITB and CD but these findings need to be validated [45]. Another report suggests that anti-I2 (Pseudomonas fluorescens-associated sequence) may also, in addition to ASCA, discriminate against GITB and CD [46]. Most studies from India do not suggest any value of ASCA testing for discriminating GITB and CD [44, 47, 48]. We do not, at present, use any of these antibodies for differentiating GITB from CD.

### Endoscopic findings

**Fig. 2:**
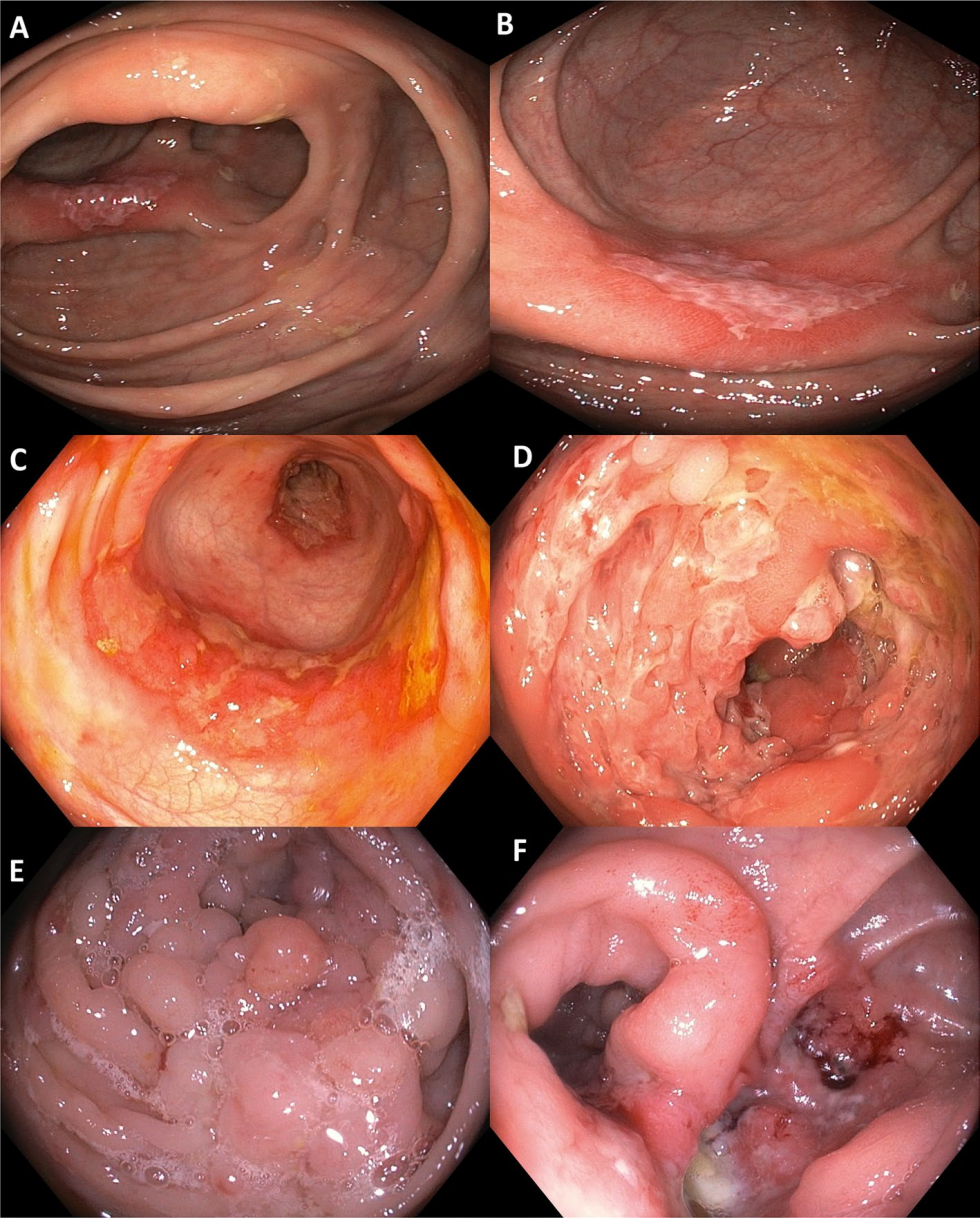
Colonoscopic images in gastrointestinal tuberculosis **A**) Caecal and **B**) Ascending colonic ulcer **C**) Transverse ulcer in ascending colon with distorted ulcerated and narrowed caecum seen in distance suggestive of skip lesions **D**) Ulcerations and pseudopylp like lesions in caecum **E**) Image of caecum in a patient treated for tuberculosis with multiple pseudoplyps and narrowing eventually requiring surgery **F**) Distored narrowed and ulcerated caecum with gaping ileocecal valve

**Fig. 3:**
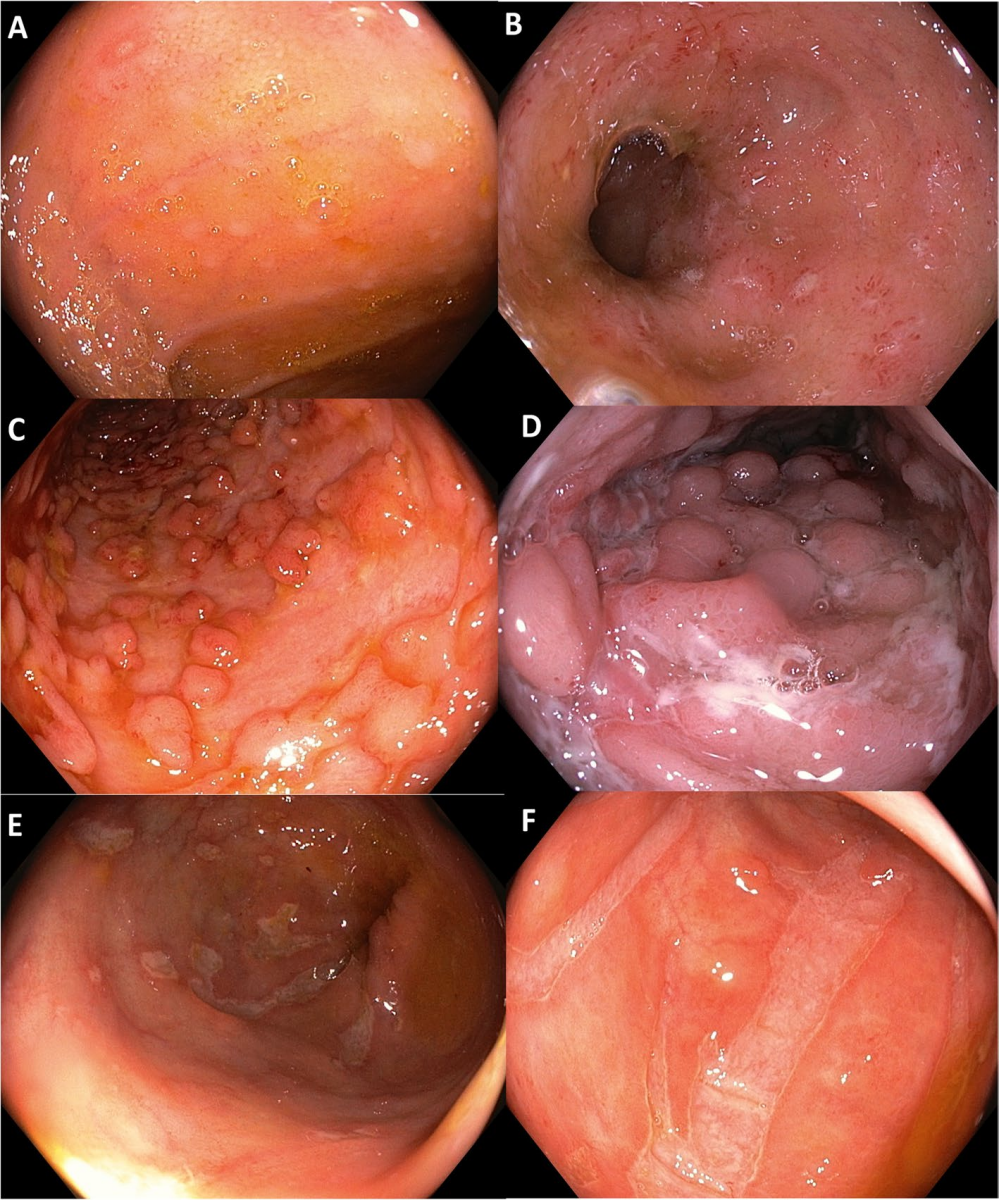
Colonoscopic images in Crohns’s disease **A**) Apthous Ulcer in terminal ileum, the patient also had multiple other ulcers on capsule endoscopy **B**) Changes of ileitis with multiple small apthuous like ulcers **C**) Deep sepiginous ulcers with pseudopylps like lesions **D**) Cobblestoning with deep intervening ulcers **E**) Left colonic ulcers of variable sizes **F**) Longitudinal parallel ulcers in colon

Endoscopy is vital for diagnosing and managing GITB and CD. Ileocolonoscopy is the procedure of choice to diagnose both GITB and CD. The type of lesions, morphological characteristics of ulcers, their location, predominant regions of involvement and additional findings like pseudopolyps, etc., may suggest the underlying diagnosis. Both GITB and CD may have ileocecal involvement, although left-sided lesions may be more suggestive of CD. Certain endoscopic features like aphthous ulcers, linear ulcers, cobblestone appearance, and skip lesions are more frequent in CD (Fig. 2). In contrast, circumferential ulcers, transverse ulcers, and patulous ileo-cecal (IC) valve are more frequent in patients with GITB (Fig. 3) [2]. None of the endoscopic features, however, are specific to each disease. Other endoscopic findings like pseudopolyps, mucosal nodularity, and stricture do not have much discriminative value [18].

CD can affect any segment of the bowel. It can be either ileal, colonic, or ileocolonic. GITB, like CD, commonly affects the ileocecal region, but colonic involvement of any region can also be seen. Apart from ulcers, GITB can have strictures, or hypertrophic lesions like polypoidal mass. Segmental ulcer or colitis is the most common form of colonic TB. However, the rectum is rarely involved in TB [32, 49]. Isolated involvement of jejunum is infrequent in patients of GITB, but jejunal involvement can be seen in patients with CD. In a prospective study that compared capsule endoscopic findings in patients with CD and GITB, involvement of the proximal segment of the bowel, multiple segment involvement, and presence of aphthous ulcers were more common in CD. Patients with GITB patients had a higher frequency of IC junction involvement [50].

The diagnostic value of colonoscopy findings was evaluated in a comparative study. Four parameters (aphthous ulcer, longitudinal ulcer, cobblestone appearance, and anorectal involvement) were more frequent in CD, and four other parameters (transverse ulcer, patulous IC valve, pseudopolyps, and less than four segment involvement) were more common in GITB [51]. Colonoscopy provides an opportunity to obtain tissue samples for histological and microbiological tests. Biopsy should be taken from the ulcer margin and sent for mycobacterial culture and molecular tests (polymerase chain ration-based tests, Nucleic acid amplification tests or NAATs) apart from routine histology [36]. The optimal number of biopsy samples needs to be standardized, but the higher the number of biopsy samples, the better the yield of the positive result for TB. Recently, Indian Council of Medical Research (ICMR), in their standard treatment workflow, has suggested taking at least six biopsy samples in sterile saline solution for microbiological analysis [52]. The endoscopic features of CD and GITB are summarised in Fig. 1.

### Imaging features

The bowel can be evaluated by plain radiographs, ultrasound (US), barium studies, computed tomography (CT), magnetic resonance imaging (MRI), and positron emission tomography (PET)-CT. The role of abdominal radiographs is limited except in emergencies like acute intestinal obstruction or suspected gastrointestinal perforation. A chest radiograph may be useful as approximately one-fourth of GITB patients may have evidence (past or current) of pulmonary TB [53]. Barium studies, including barium meal follow-through (BMFT) and enteroclysis, although popular in the past, are not frequently performed now. This is because of declining expertise in interpreting barium studies, higher radiation exposure, and also because of advances in cross-sectional imaging (CT/MR enterography and enteroclysis), which have allowed simultaneous assessment of luminal and extraluminal pathology. Typical findings on a BMFT in GITB include linear or transverse ulcers in the terminal ileum, short segment strictures, and contracted and pulled-up caecum. Fleischner’s sign due to patulous and gaping IC valve associated with narrowing of the terminal ileum, and Steirlin sign, which is rapid emptying of barium from the caecum to ascending colon due to irritable caecal mucosa may be seen in GITB. The other features are purse string stenosis opposite to the IC valve associated with dilated terminal ileum [54]. On the contrary, BMFT in patients with CD shows aphthous ulcerations, deep ulcerations, longitudinal ulcerations, cobblestone appearance, and fistulas. There may be sacculations on the antimesenteric border because of the shortening of the mesenteric border as longitudinal ulcers heal by fibrosis. Involvement of multiple segments of the bowel along with normal intervening segments are characteristic of CD. Although the findings mentioned above are characteristic of CD, none are pathognomonic. The sensitivity of BMFT to detect CD is 67–72% [55].

Ultrasound (US) is an inexpensive and readily available modality for assessing bowel diseases. Recently, there has been considerable interest in intestinal ultrasound for IBD. The US findings in CD include an increase in bowel wall thickness (> 3 mm), an increase in colour doppler signal due to mucosal inflammation, and increased hyperechogenicity in mesenteric fat and regional lymphadenopathy [56]. US is also an excellent initial modality for evaluation in suspected GITB. It can identify bowel thickening (including the ileocecal region), abdominal lymphadenopathy, the presence of ascites, omental or peritoneal changes which could be part of tubercular pathology [57]. Further, US can help acquire tissue from lymph nodes, and peritoneal or bowel pathology either by fine needle aspiration or core biopsy. The limitations of the US include problems in assessment in the presence of bowel gas, obesity, and the radiologist's experience. The major role of bowel US is to assess the disease activity and to see the treatment response by using a color flow doppler in the bowel wall.

**Fig. 4:**
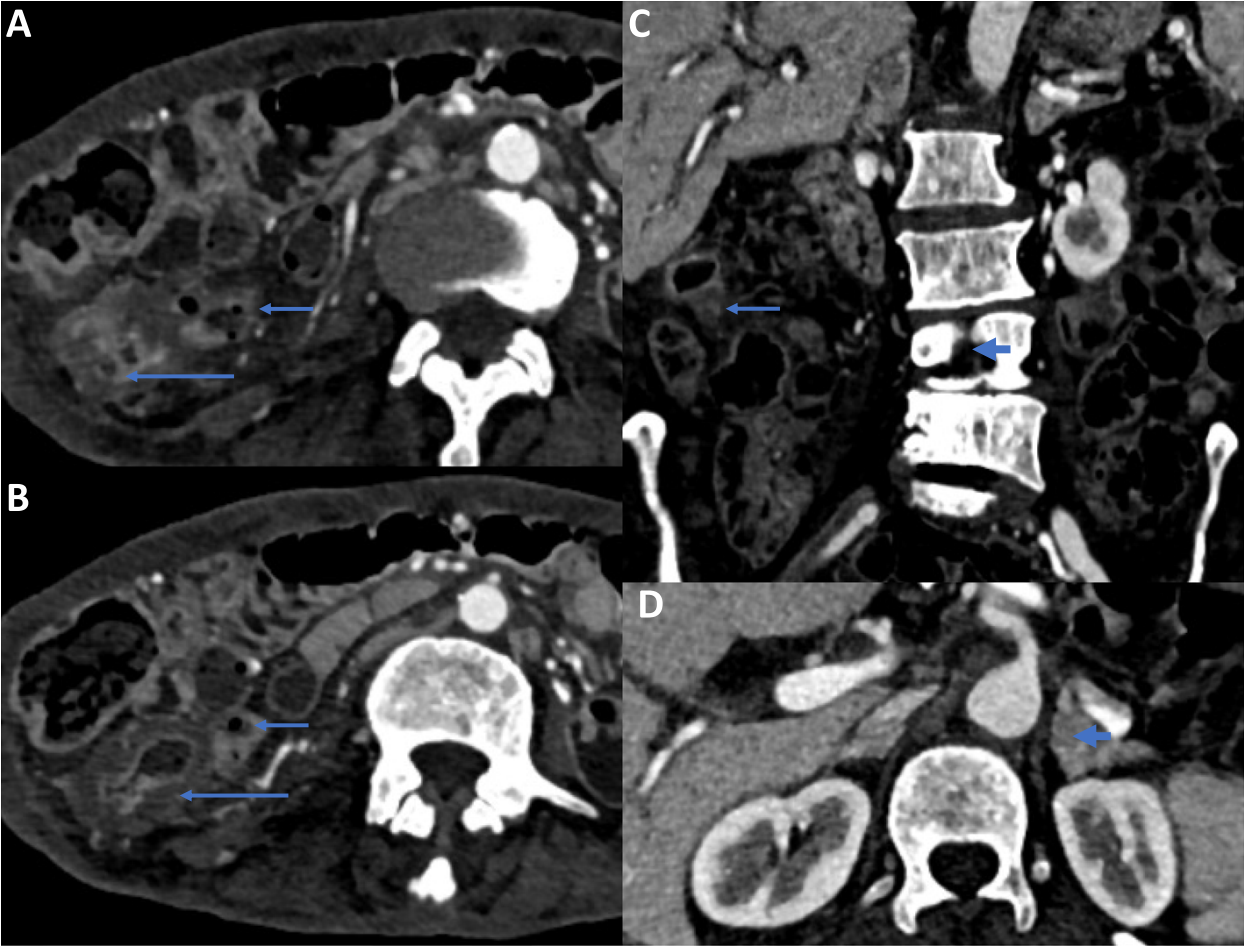
Axial (**A**, **B**) and coronal (**C**) contrast enhanced CT enterography images show contiguous asymmetric circumferential mural thickening of the caecum (arrows, **A** and **B**) and terminal ileum (short arrows, **A**-**C**). The caecum is pulled up in subhepatic location (not shown). Also note the lumbar vertebral lesion (arrow head). Axial CT image at the level of adrenals shows bulky and heterogeneous left adrenal gland (arrow head) suggestive of granulomatous involvement. The right adrenal gland was also involved (not shown). The patient was diagnosed to have disseminated tuberculosis

Contrast-enhanced CT of the abdomen and CT-enterography (CTE) have the advantage of evaluating both intestinal and extraintestinal lesions, detecting early changes and subtle changes of GITB and CD, and potentially differentiating active disease from fibrotic disease. In patients with active GITB, there is circumferential wall thickening and mucosal enhancement in the terminal ileum and ileocaecal region. Mucosal enhancement in patients with GITB is homogenous and usually without any stratification [58]. The healed disease may show short segment stricture without mucosal enhancement and stratification [58]. CT can also detect extraintestinal findings in patients with GITB, including enlargement of mesenteric and retroperitoneal lymph nodes, ascites, omental or peritoneal thickening, abdominal cocoon, or the involvement of other solid organs like the liver or spleen (Fig. 4). Enlargement of the lymph nodes (typically > 1 cm) in patients with tuberculosis can be discrete or conglomerate and often necrotic. Necrotic lymph nodes are specific for GITB.

**Fig. 5:**
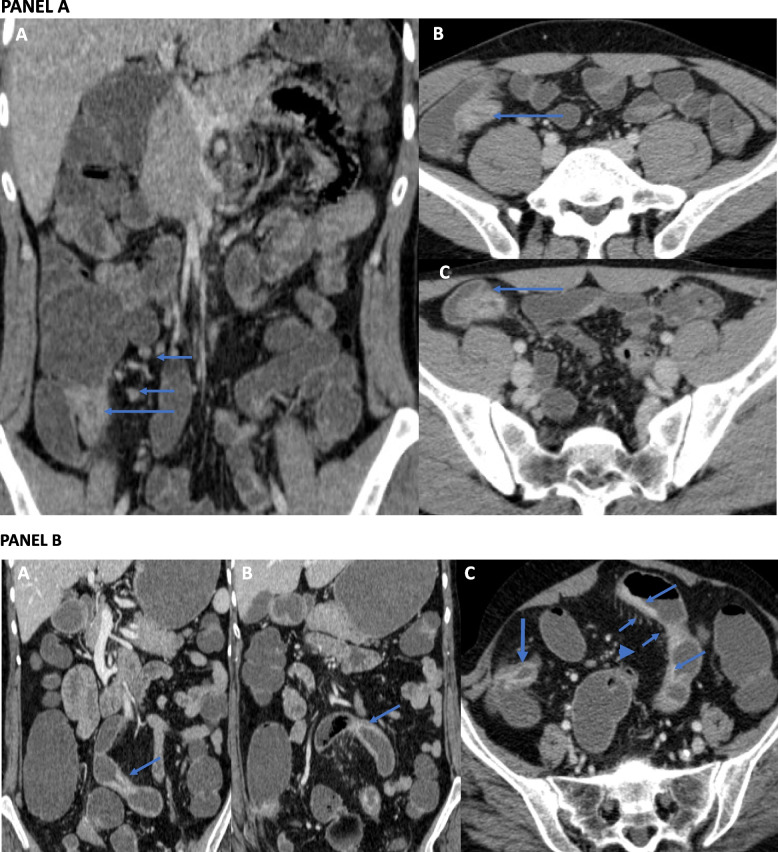
:PANEL **A**: Coronal (**A**) and Axial (**B**, **C**) contrast enhanced CT enterography images show circumferential mural thickening of the terminal ileum and ileocaecal junction (arrows). There are subcentimetric mesenteric lymph nodes (short arrows, **A**). The patient was diagnosed to have Crohn’s disease. PANEL **B**: Coronal (**A** and **B**) and Axial (**C**) CT enterography images show multisegmental asymmetric mural thickening of the ileum (arrows). Note the engorged vasa rectae (short arrows, **C**) and mesenteric fat proliferation (arrow head, **C**). The terminal ileum is also involved (thick arrow, **C**) and shows mural stratification. The patient was diagnosed to have Crohn’s disease

The typical findings of CD on CTE are asymmetrical circumferential wall thickening in the terminal ileum and caecum with greater involvement in the terminal ileum than the caecum. Active disease is characterized by mucosal enhancement and mural stratification. Mesenteric changes include prominent vasculature in the mesentery close to a thickened bowel loop (Comb sign), fibrofatty proliferation, and mesenteric fat stranding (Fig. 5) [59]. CT can also detect the fistulas that are more common in CD than GITB, and can be entero-enteric, entero-colic, colo-colic, or perianal. Fistula with other viscera (e.g. entero-vesical, entero-vaginal) can also be seen. The extraintestinal changes of CD are intrabdominal abscess complicating CD and mesenteric changes [60]. In a systematic review and meta-analysis of CT features to differentiate GITB from CD, necrotic lymph nodes had a pooled sensitivity of 23% and specificity of 100% to diagnose GITB, while Comb sign and skip lesions have a sensitivity of 82% & 86% and specificity of 81% & 74%, respectively to diagnose CD [33].

The changes seen at MR enterography (MRE) are similar to those seen at CTE. MRI has a greater soft tissue resolution and the advantage of being non-ionizing. Additionally, MR sequences can differentiate active inflammatory disease from fibrotic disease. Fibrotic strictures are hypointense on the T2-weighted MRI image and demonstrate loss of stratification without diffusion restriction [61].

The role of newer image imaging techniques like contrast-enhanced intestinal ultrasonography, small intestine contrast ultrasound, dynamic contrast enhancement (DCE) MRI, quantification of diffusion restriction by calculating apparent diffusion coefficient (ADC), and hybrid PET/MRI needs evaluation for discriminating GITB from CD [57]. More recently, in an Indian study, certain parameters like blood flow, permeability and mean transit time as assessed on perfusion-CT could differentiate CD from GITB [62].

The target of therapy for both GITB and CD is mucosal healing, and that can potentially also be assessed with the help of radiology. In fact, in the last decade, radiology, primarily intestinal ultrasonography (IUS), has shown promising results both in diagnosing and monitoring responses to therapy in CD. In a systematic review and meta-analysis of 39 studies on trans-abdominal ultrasound in response assessment in IBD, IUS had comparable efficacy to endoscopy and MRE. The parameters assessed for response definition on IUS were a reduction in bowel wall thickness > 25% from baseline or > 2 mm or > 1 mm with one color doppler signal reduction [63]. IUS has another advantage over ileocolonoscopy that it can assess the transmural healing of the lesion. In a prospective study of 77 patients with CD mucosal healing was assessed both with IUS for transmural healing and endoscopy for mucosal healing, and they observed that achievement of transmural healing has a better long-term outcome [64]. Similarly, IUS has also been studied for response assessment to ATT for GITB. In a retrospective study of 20 patients with GITB on ATT, response to therapy is assessed with IUS based on bowel wall morphology and Limberg score, and they observed that the sensitivity and specificity of IUS for evaluation to response to ATT was 100% and 50% respectively [65]. Use of MRI-based parameters has also been reported- a significant rise in apparent diffusion coefficient was noted in those who had a response to ATT [66]. It is unclear if these changes are also detectable early after the initiation of ATT.

In summary, based on the site and pattern of involvement, and the presence of sinuses or fistulas, necrotic lymph nodes, and other ancillary findings, imaging studies help differentiate GITB from CD. Caecum and right-sided colon are more involved in GITB (83%) than CD (33%). Left-sided colon is more frequently involved in CD than GITB. More patients with CD have skip lesions (99%) than GITB (15%) [3]. Asymmetric and greater (more than 6 mm) bowel wall thickness and wall stratification are seen more commonly with CD than GITB [67].

Eccentric stricture with sacculation is usually seen with CD, while concentric strictures are more common in GITB. Peritoneal thickening, omental caking, ascites, cocoon formation, and the necrotic mesenteric lymph node highly suggest GITB, and enteroenteric fistula, mesenteric fibrofatty proliferation, small homogenous mesenteric lymph node, and perianal fistulas are more characteristic of CD. Figure 1 summarises the radiological differences between GITB and CD.

### Histopathology

Distinguishing GITB and CD is challenging due to the overlapping macroscopic and microscopic histological features. Endoscopic biopsy has, however, become an important diagnostic tool to shed light on the few mucosal differences present in both diseases making differentiation possible. Granuloma detection rates in intestinal biopsies vary in the literature ranging from 10 to 80% in GITB cases and 15% to 65% in CD patients [36, 68]. As expected, the yield of endoscopic mucosal biopsies is lower than surgically resected specimens. Obtaining a reliable diagnosis requires an appropriate number of mucosal biopsies. According to the European Crohn’s and Colitis Organization and the European Society of Pathology, multiple biopsies from five sites around the colon (including the rectum) and ileum should be obtained for an adequate histopathological diagnosis of inflammatory bowel disease. Multiple biopsies (minimally two samples from each side of the intestinal wall) show a better understanding of the distribution pattern of the inflammation than single tissue biopsies, secondarily increasing the granuloma detection rate. The highest granuloma detection rate can be established in cases showing longitudinal ulcers on endoscopy [69]. In one study, basal plasmacytosis was exclusively detected in cases with longitudinal ulcer and cobblestone appearance [69].

GITB and CD show both macroscopic and microscopic differences. Macroscopic features of GITB are short strictures, marked inflammatory thickening, fibrosis, and adhesions. Ulcerations are also a prominent feature of GITB and are transverse and often circumferential with ill-defined, sloping, or overhanging edges. The surrounding mucosal areas show changes like flattened mucosal folds, ulcerations, erosions, and pseudopolyps. Cross section of the intestinal wall often shows a loss of distinction between the different intestinal wall layers due to scar tissue and necrosis. Multiple 2–5 mm nodules and adhesions can be present within the serosa. Furthermore, GITB patients show enlarged regional lymph nodes with possible caseation [70].

Like GITB, CD also shows bowel wall thickening and strictures, although these are typically longer. Additionally, CD patients often present with adhesions, fistulae, sinuses and extra-intestinal abscesses. Different types of ulcerations can be seen in CD ranging from deep longitudinal fissuring ulcers to smaller longitudinal ones separating oedematous or unaffected parts of the mucosa creating a cobblestone pattern. Ulcers are seen to change with disease stages, appearing small aphthous in the initial phases and coalescing, and evolving to larger stellate ulcers [71, 72].

**Fig. 6:**
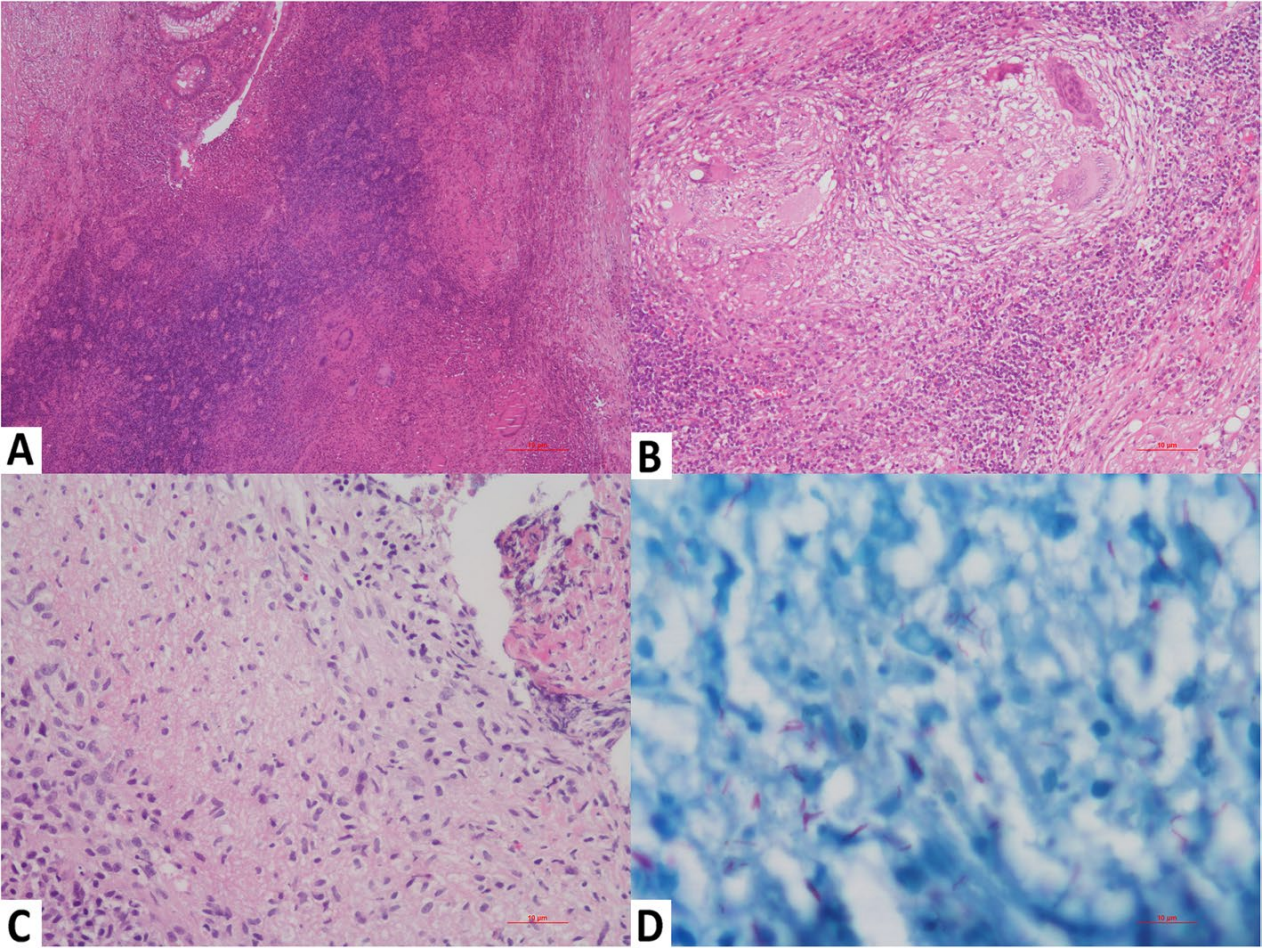
Histopathological examination of resected ileocecal specimen depicting many epithelioid cell granulomas throughout the intestinal wall (**A**, Hematoxylin and Eosin stain, 40x). Epithelioid cell granulomas are large with lymphocytic cuffing and Langhan’s type multinucleate giant cells (**B**, Hematoxylin and Eosin stain, 200x). Microscopy of ileal biopsy of one of our case showing ulceration of lining with many epithelioid histiocytes, necrosis (**C**, Hematoxylin and Eosin stain, 200x) and numerous acid fast bacilli (**D**, Ziehl Neelsen’s stain, Oil immersion)

Granulomas are vaguely defined collections of epithelioid histiocytes or macrophages and a feature of both GITB and CD. Multiple (5 or more granulomas per hpf), large ((> 200–400 μm), confluent caseating granulomas with acid-fast bacilli surrounded by a lymphoid border are characteristic of GITB. In GITB, the granulomas can typically be found in all layers of the intestinal wall and/or inside lymphoid tissue. Early granulomas are frequently found within the latter. Sometimes even extensive pyloric metaplasia can be present. Healing granulomas are characterized by fibrosis and epithelial regeneration emerging at the edge of ulcers. A circumferential fibrous cuff around healing granulomas is exclusively seen in lymph nodes and not in the intestinal mucosa (Fig. 6) [70–72].

**Fig. 7:**
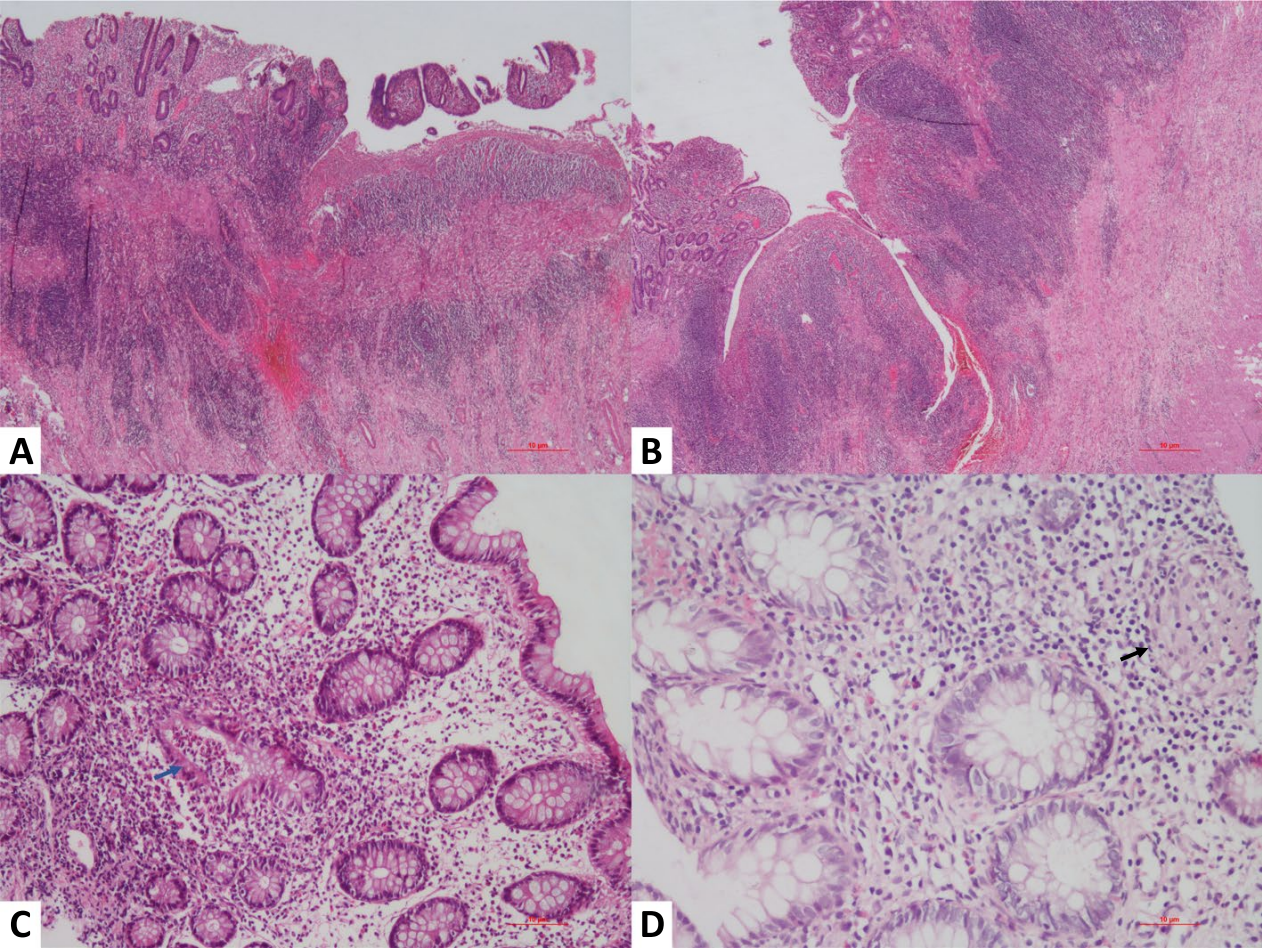
Histopathological examination of resected colon showing skip ulcers alternating with intact mucosa and transmural dense inflammation (**A**, Hematoxylin and Eosin stain, 100x). Fissuring ulcer are extending up to submucosa with numerous lymphoid follicles (**B**, Hematoxylin and Eosin stain, 100x). Microscopy of intestinal biopsies showing active colitis features such as cryptitis and crypt abscesses focally (**C**, Hematoxylin and Eosin stain, 200x) and microgranulomas in the lamina propria (**D**, Hematoxylin and Eosin stain, 200x)

On the other hand, common microscopic features in CD include deep fissuring ulcers extending into the muscularis propria or beyond, distortion of the mucosal architecture, pyloric metaplasia, cryptitis with abscess formation, and moderate to severe chronic inflammation. The ulcerations show a segmental to patchy distribution, often extending transmurally. The granulomas are relatively small (microgranulomas), discrete, less frequent and are found in 50–60% of resected intestinal material [3, 36, 68, 73]. Microgranuloma refers to small, poorly organized collections of histiocytes in CD, and such microgranulomas are observed in 10% of the biopsy specimens from endoscopically uninvolved mucosa, along with chronic inflammatory changes in 71% of such sites. Additionally, in 25% of the cases, the granulomas were located in regional lymph nodes (but almost never without intestinal involvement). Prominent lymphoid follicles in the submucosa and serosa are an additional characteristic feature in patients with CD (Fig. 7) [71, 72].

Granulomas in the surrounding intestinal lymph nodes can be present in both GITB and CD, but their presence in the absence of intestinal inflammation is exclusively related to GITB [73]. However, excessive disproportional submucosal inflammation is common in GITB. GITB shows more submucosal granulomas and ulcers lined by macrophages and has a higher prevalence of deep ulcers. Other common histological characteristics of CD include focal-enhanced colitis and architectural distortion at sites distant from granulomatous inflammation, like crypt distortion, branching, shortening, decreased crypt density, and irregular mucosal surface. In a retrospective study, focal lesions were also seen in the biopsy specimens of GITB, other infections, ischaemic colitis, and partially treated ulcerative colitis [68, 73].

According to a meta-analysis including 316 GITB patients and 376 CD patients, three histological features were found to have a high specificity for diagnosing GITB compared to CD. These included caseating necrosis, confluent granulomas, and macrophage-lined ulcers with a pooled sensitivity of 21%, 38%, and 41%, and a pooled specificity of 100%, 99%, and 95%, respectively in the differentiation between GITB and CD. These histological features have poor sensitivity, although excellent specificity [74]. Further, surgical biopsies frequently show transmural fissures extending to the serosa in cases of CD, whereas they are rarely seen in GITB [5].

There have been attempts to differentiate between both diseases through immunohistochemistry (IHC). An Indian study reported high diagnostic specificity for CD-73 in tuberculous granulomas in GITB, along with the finding of distinguishable markers of MSC CD29, CD90, and CD150 [25].However, a South African study showed that CD73 positivity was found in 52% of GITB patients and 30% of the CD cohort, disproving the exclusivity of CD73 positivity in GITB [75]. Therefore, conclusive histopathological diagnosis is often not possible. These studies have emphasized the diagnostic difficulties in distinguishing these two conditions, even with the availability of surgically resected specimens [73].

### Microbiology

Microbiological positivity, through acid fast-bacillus (AFB), culture, or polymerase chain reaction (PCR) based techniques, is usually considered a gold standard for diagnosing GITB. However, microbiological techniques, have a with very low sensitivity.

Microbiological positivity is the gold standard for diagnosing GITB. Unfortunately, the AFB positivity rate in the intestinal tissue is very low compared to sputum, limiting its role in diagnosing GITB. The sensitivity of AFB positivity in intestinal tissue in GITB is less than 5% [14]. However, mycobacterial culture can increase the yield of diagnosing GITB and provide the opportunity for drug sensitivity. The reported rate of culture positivity in the literature is 7–79% [53]. The traditional culture done with the Lowenstein-Jensen medium is time-consuming. Mycobacterium Growth Indicator Tube 960 (MGIT) provides quick results with better sensitivity than traditional culture. The sensitivity of MGIT to diagnose GITB is 40–52.8% [76].

PCR-based tests help provide quick diagnosis and are based on the principle of DNA extraction, DNA amplification, and DNA detection. Usually, the target sequence for amplification is IS6110 which is more specific to *Mycobacterium tuberculosis*. In a systematic review and meta-analysis of 9 studies to assess the diagnostic value of MTB-PCR (IS6110) to diagnose GITB and to differentiate it from CD, a pooled sensitivity of 47% and a pooled specificity of 95%was reported [77]. As it has high specificity and low sensitivity, and many Indian strains do not have this gene sequence, thus limiting its sensitivity in the diagnosis. Another point of care platform for rapid diagnosis of GITB is the Xpert-Mtb/Rif. A systematic review and meta-analysis of five studies assessed the diagnostic accuracy of XpertMtb/Rif for diagnosing GITB in intestinal tissue and observed a pooled sensitivity of 23% and a pooled specificity of 100% to diagnose GITB in intestinal tissue [78]. In an Indian study, multiplex PCR utilizing three genes IS6110, MPB64, and Protein B, showed high sensitivity and specificity to diagnose GITB. Chip-based real-time PCR assay (TrueNAT MTB Plus) has the advantage of quick diagnosis and drug sensitivity testing. An Indian study evaluated the performance of Truenat MTB plus and reported that the sensitivity and specificity of Truenat MTB plus to diagnose GITB were 70% and 100% [79]. However, this report was based on intestinal tissue testing of previously diagnosed cases, and real-life studies are required to define the place of Truenat in the differentiation of GITB and CD. When combined with histopathology, the multiplex PCR test can detect 97.5% of GITB [80]. No data is available regarding newer PCR-based tests like Xpert-Ultra.

### Models based on multiple parameters

As is apparent from the preceding text, although certain features suggest one diagnosis over the other, none of the clinical, endoscopic, and laboratory parameters are exclusive to either GITB or CD. Multiple attempts have been made to combine and incorporate multiple features in models or nomograms for discriminating GITB and CD [18, 32, 51, 81–92].

A landmark study by Limsrivilai et al. using a Bayesian model observed that gender, clinical manifestations, endoscopic features, and laboratory findings can accurately diagnose GITB in 91.8% of patients. This model has a sensitivity of 90.9% and a specificity of 92.6% for diagnosing GITB. [18] The model requires the imputation of baseline prevalence of the disease. The validation study by the same author reported that the model outperformed three other models in a five-center study. Also, it had the lowest rate of GITB being misdiagnosed as CD [93]. Another Indian study using clinical, endoscopic, and histological findings created a multivariate logistic model. Using four variables (presence of blood in stool, weight loss, involvement of the sigmoid colon, and focally enhanced colitis), a final score was calculated, which ranged from 0.3 to 9.3. The higher the score is, the higher the chance of GITB. With a cut-off of 5.1, the model has a sensitivity of 83.0%, a specificity of 79.2%, and an accuracy of 81.1% in classifying the two diseases [32]. Li et al. analyzed clinical and endoscopic features in their retrospective study by logistic regression analysis. They reported that the presence of haematochezia, history of intestinal surgery, presence of perianal disease and pulmonary tuberculosis, presence of ascites, and positive Mantoux test have a sensitivity of 90.3% and specificity of 76.8% to differentiate GITB from CD. They reported that the involvement of rectum, longitudinal ulcer, and cobblestone appearance, has high accuracy in predicting CD while transverse ulcer, fixed open IC valve, and rodent ulcer can predict GITB. [81] A study from China included night sweats, the presence of a longitudinal ulcer, and the granuloma in their diagnostic model; to differentiate GITB from CD with good diagnostic accuracy [82]. Another Korean study used colonoscopy, radiological, and laboratory parameters (ASCA and IGRA) in their model, and they reported accuracy of the model was 96% to differentiate GITB from CD [90].

Various multiparametric models have been studied in various populations to differentiate GITB from CD. However, all of the studies have limitations by small sample size, lack of validation cohort (except the Limsrivilai model), and use of complex formulas to calculate the score, thus limiting its use in day-to-day practice. Table 1 summarizes various multiparametric-based predictive models to differentiate GITB from CD.

**Table 1:** Different multiparametric predictive model to differentiate GITB from CD

Author	Year	Study	Clinical	Endoscopic	Histology	Imaging	Laboratory	Sensitivity	Specificity	AUROC	Validation Cohort
Makharia et. al [32]	2010	Prospective	Yes	Yes	Yes	No	No	83%	79%	0.91	Yes
Li et. al [81]	2011	Retrospective	Yes	Yes	No	Yes	Yes	83%	82%	0.83	No
Yu et. al [82]	2012	Retrospective	Yes	Yes	Yes	No	No	67%	93%	0.86	No
Zhao et. al [86]	2014	Retrospective	Yes	No	No	Yes	No	94%	80%	0.96	No
Mao et. al [84]	2015	Prospective	No	Yes	No	Yes	No	93%	80%	0.86	Yes
Zhang et. al [87]	2015	Retrospective	Yes	Yes	No	Yes	No	98%	97%	0.99	No
Huang et. al [89]	2015	Retrospective	Yes	Yes	No	Yes	Yes	100%	95%	0.99	No
Jung et. al [83]	2016	Retrospective	Yes	Yes	No	No	No	98%	92%	0.98	Yes
Bae et. al [90]	2017	Prospective	No	Yes	No	Yes	Yes	95%	97%	0.96	Yes
Wu et. al [91]	2018	Prospective	Yes	Yes	No	No	Yes	89%	93%	0.95	yes
Limsrivilai et. al [18]	2017	Retrospective	Yes	Yes	Yes	Yes	Yes	91%	93%	0.94	Yes
He et. al [92]	2019	Prospective	Yes	Yes	No	Yes	Yes	87%	91%	0.97	Yes
Watermeyer et. al [1]	2018	Retrospective	Yes	No	Yes	Yes	Yes		93%	0.88	No

### Newer testing methods and use of artificial intelligence

Mesenteric fat is believed to be the driving force of inflammation in CD [94]. Quantifying visceral fat and its ratio to total fat or subcutaneous fat has been evaluated in numerous studies to differentiate CD from GITB. A retrospective study from South Korea first reported that the ratio of visceral fat (VF) to total fat (TF) and visceral fat (VF) to subcutaneous fat (SF) is significantly higher in patients with CD than in patients with GITB. They also report that the VF/TF value of 0.46 has a sensitivity of 42.1% and a specificity of 93.3% to diagnose CD [95]. These findings were confirmed by two later studies from India [96, 97]. One of these studies suggested that a combination of two radiological findings i.e. increased visceral fat and a long segment (> 4 cm) involvement of the bowel wall, had high specificity for the diagnosis of CD [97].

A dysregulated immune response characterizes CD. A dysfunction in the number, function, and ability of the regulatory T cell (CD4 + CD25 + FOXP3 +) to home to the gut mucosa is recognised. The level of these FOXP3 + -T regulatory cells is low in patients with CD and high in patients with GITB. A prospective study from India reported that a cut-off value of > 32.5% for FOXP3 + -T reg cell in peripheral blood could differentiate GITB from CD with 70% sensitivity and 90.6% specificity [98]. Serum cytokine levels do not seem to discriminate the two entities [99]. The pattern of polarization of macrophages on activation can also help in differentiating GITB from CD. A study using IHC-based staining of colonic biopsies from patients with CD and GITB observed that M1 polarization is noted more frequently with CD and M2 polarization is more frequent in GITB. Even in granuloma-positive biopsy samples in patients with CD, M1 polarization of macrophage is significant [100].

CD and GITB may have unique pathophysiological signatures. With the help of proteomics using high throughput technologies, it could be possible to characterize various proteins and their structure and associated post-translational modification. Matrix- assisted laser desorption ionization – time of flight (MALDI-TOF) is a mass spectrometry technique that can be used on serum samples to identify differentially expressed. The two protein peaks, appetite peptide and lysyl oxidase-like 2 (LOXL-2), have been studied to differentiate GITB from CD [101]. Similarly, another proteomics study to identify differentially expressed proteins using tandem mass tag-labelled proteomics technology identified 108 differently expressed proteins to differentiate GITB from CD [102]. Analysis of tissue proteomics using liquid chromatography-mass spectrometry has also been studied. However, in a validation study using immunohistochemistry, none of the differently expressed proteins could discriminate GITB from CD [103]. Other approaches have been assessed with not-so-promising outcomes. A study reported a Liquid chromatography–mass spectrometry-based approach on mucosal proteomics where they distinguished 11 proteins that are differentially expressed in the GITB and CD cases. However, in an attempt to replicate these findings in another cohort of patients, six proteins were not expressed differently in GITB and CD [2].

Masson’s trichrome staining and second harmonic generation, and two-photon excited fluorescence imaging could be used in the differentiation. Using these techniques, a recent study showed that collagen fiber and fiber deposits in intestinal biopsies are significantly higher in GITB than in CD [104].

Most multiparametric models use logistic regression to calculate a score that provides the continuous probability of GITB or CD. But they are limited by the inability to solve the nonlinear interaction between the variables and failure to solve the problem of imbalance. Machine learning algorithms can overcome the limitation of statistical methods and produce superior out-of-sample performance. There are multiple reports which have utilized various AI-based approaches. These have utilized reporting text, endoscopic, and radiological images in an attempt to make a distinction but await validation [4, 105–108].

### Response to antitubercular therapy

Even using all available armamentaria, differentiating GITB and CD may not be possible in a subset of patients. A therapeutic trial of Anti-Tubercular therapy (ATT) is one of the oldest methods to diagnose GITB and to differentiate GITB from CD [109]. However, it requires an appropriate, objective, and timely assessment of the response to ATT. The reverse approach of treating with steroids or immunosuppression is not usually preferred in TB-endemic regions because of the lack of clear endpoints and the risk of TB dissemination with immunosuppression [30]. The use of ATT first, although preferred, is not without risks- ATT-induced hepatitis and delay in the diagnosis of CD resulting in stricturing disease and increased risk of surgery is well recognized.

The legendary work of Logan elucidated the definition of response to ATT in anorectal TB by looking at the healing of the lesions. It should be noted that assessment of clinical responses alone may be misleading- many patients with CD have symptomatic improvement and reduction in inflammatory markers like CRP [21, 22]. Further, even patients with GITB could continue to be symptomatic due to underlying stricturing disease [110]. Therefore, clinicians should seek more specific responses which are unequivocal. In this regard, mucosal healing of the ulcers while on ATT is considered the gold standard as a surrogate for underlying GITB [21]. Early mucosal response to therapy by doing a colonoscopy at two months of ATT not only helps to differentiate GITB from CD but also could prevent the development of complicated CD by allowing timely initiation of therapy for CD [111]. Early reassessment provides a surety of diagnosis and an opportunity for early evaluation of the reasons behind the lack of response, including multidrug-resistant GITB or, more frequently, Crohn’s disease. However, most MDR-TB patients also have associated pulmonary tuberculosis [112].

Non-invasive markers have also been studied as response criteria to ATT. An Indian study by Sharma et al., observed that serial monitoring of CRP at baseline and two months helps evaluate response to ATT. Normalization of CRP at two months was predictive of mucosal response to ATT. [22] They also studied the combination of serum CRP with fecal calprotectin to assess response to ATT. They observed that fecal calprotectin is a better marker of mucosal healing than CRP because some decline of CRP with ATT also occurs in CD [23]. The use of radiology (including ultrasound and MRI) to assess response to ATT has been summarised in the section on Imaging.

### Guidelines

In response to this diagnostic dilemma, the Asia–Pacific guidelines in 2016 recommend 8–12 weeks of empirical antituberculosis treatment (ATT) for patients with diagnostic uncertainty due to the possible onset of potentially fatal complications if immunosuppressive agents are inappropriately prescribed to GITB patients [113]. It is also emphasized that all patients on the ATT trial should undergo repeat colonoscopy and biopsy at 8- 12 weeks if there is minimal or no response to therapy. Those with a complete or partial response to ATT should undergo a colonoscopy at six months to document mucosal healing. The Asia–Pacific guideline does not recommend starting concomitant treatment for GITB and CD except when the patient needs immediate therapy for severe disease. The concomitant therapy for both GITB and CD will create a diagnostic dilemma on a long-term basis. However, 8–12 weeks of empiric ATT can delay appropriate CD treatment, leading to exacerbation and disease-related complications [6]. In 2020, the Asian organization for Crohn’s colitis and the Asia Pacific Association of gastroenterology recommended that GITB should be ruled out even before the diagnosis of IBD and, if necessary, a diagnostic ATT trial can be started in complex cases. The persistence of symptoms at three months of ATT favours the diagnosis of CD [114]. In 2021 World Gastroenterology Organisation Global Guidelines suggest using empiric ATT for 2–3 months and weekly response assessment. Assessment of response is carried out by resolution of symptoms and gaining of weight [115]. The diagnosis of GITB is likely if there is a complete response of symptoms and no relapse in follow-up. However, we suggest that all patients, irrespective of clinical response, should undergo repeat colonoscopy at two months if started on empirical ATT (diagnostic trial of ATT). This is because, as mentioned earlier, the clinical responses to ATT can be misleading in patients with CD and GITB. While patients with CD could have a symptomatic response to ATT in around a quarter of patients, patients with GITB could continue to be symptomatic due to strictures.

## Conclusion

**Fig. 8:**
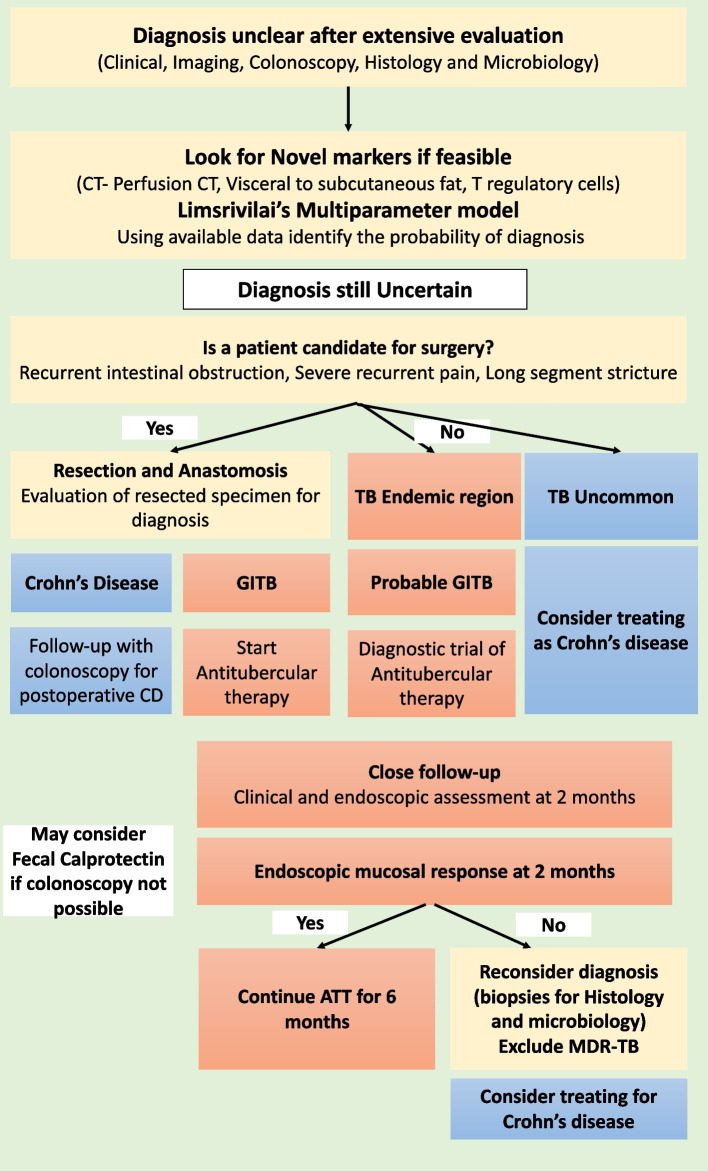
Flow chart showing an algorithmic approach to diagnosis and followup in patients with diagnostic confusion between gastrointestinal tuberculosis and Crohn’s disease even after standard evaluation

Differentiating GITB from CD is a perplexing issue faced by clinicians across the globe. In this review, we have summarised the evidence base which could help in discriminating between these two entities and also highlighted ongoing research which might improve our discriminative ability. Figure 8 shows the flow chart which we use in discriminating GITB and CD in TB endemic regions if the diagnosis remains uncertain after standard clinical, endoscopic, radiological, histopathological, and microbiological work-up. In the wake of the published evidence, we provide an algorithmic approach to differentiate the two conditions (Fig. 8), which is especially valid in TB-endemic regions. We also suggest the approach to follow-up after starting a diagnostic trial of ATT in cases with unresolved dilemma. The increasing incidence of CD in the developing world and the increasing incidence of GITB in the developed world will continue to present challenges to astute clinicians, and an evidence-based approach using objective parameters in a timely manner is important for diagnosis and response assessment.

## Data Availability

Not applicable.

## References

[1] Watermeyer G, Thomson S (2018). Differentiating Crohn's disease from intestinal tuberculosis at presentation in patients with tissue granulomas. S Afr Med J.

[2] Sharma V (2020). Differentiating intestinal tuberculosis and Crohn disease: Quo Vadis. Expert Rev Gastroenterol Hepatol.

[3] Sharma R, Madhusudhan KS, Ahuja V (2016). Intestinal tuberculosis versus crohn's disease: Clinical and radiological recommendations. Indian J Radiol Imaging.

[4] Weng F, Meng Y, Lu F (2022). Differentiation of intestinal tuberculosis and Crohn's disease through an explainable machine learning method. Sci Rep.

[5] Kedia S, Das P, Madhusudhan KS (2019). Differentiating Crohn's disease from intestinal tuberculosis. World J Gastroenterol.

[6] Zhao Y, Xu M, Chen L, Liu Z, Sun X (2020). Levels of TB-IGRA may help to differentiate between intestinal tuberculosis and Crohn's disease in patients with positive results. Therap Adv Gastroenterol.

[7] Liu JZ, van Sommeren S, Huang H (2015). Association analyses identify 38 susceptibility loci for inflammatory bowel disease and highlight shared genetic risk across populations. Nat Genet.

[8] Huang C, Haritunians T, Okou DT (2015). Characterization of genetic loci that affect susceptibility to inflammatory bowel diseases in African Americans. Gastroenterology.

[9] Mahid SS, Minor KS, Soto RE, Hornung CA, Galandiuk S. Smoking and inflammatory bowel disease: a meta-analysis [published correction appears in Mayo Clin Proc. 2007 Jul;82(7):890]. Mayo Clin Proc. 2006;81(11):1462–1471. 10.4065/81.11.1462 17120402

[10] Ungaro R, Bernstein CN, Gearry R (2014). Antibiotics associated with increased risk of new-onset Crohn's disease but not ulcerative colitis: a meta-analysis. Am J Gastroenterol.

[11] Cornish JA, Tan E, Simillis C, Clark SK, Teare J, Tekkis PP. The risk of oral contraceptives in the etiology of inflammatory bowel disease: a meta-analysis. Am J Gastroenterol. 2008;103(9):2394–2400. 10.1111/j.1572-0241.2008.02064.x 18684177

[12] Torres J, Mehandru S, Colombel JF, Peyrin-Biroulet L (2017). Crohn's disease. Lancet.

[13] Eglinton TW, Barclay ML, Gearry RB, Frizelle FA (2012). The spectrum of perianal Crohn's disease in a population-based cohort. Dis Colon Rectum.

[14] Jha DK, Pathiyil MM, Sharma V (2023). Evidence-based approach to diagnosis and management of abdominal tuberculosis. Indian J Gastroenterol.

[15] Aswani-Omprakash T, Sharma V, Bishu S (2021). Addressing unmet needs from a new frontier of IBD: the South Asian IBD Alliance. Lancet Gastroenterol Hepatol.

[16] Kaplan GG, Windsor JW (2021). The four epidemiological stages in the global evolution of inflammatory bowel disease. Nat Rev Gastroenterol Hepatol.

[17] Singh P, Ananthakrishnan A, Ahuja V (2017). Pivot to Asia: inflammatory bowel disease burden. Intest Res.

[18] Limsrivilai J, Shreiner AB, Pongpaibul A (2017). Meta-Analytic Bayesian Model For Differentiating Intestinal Tuberculosis from Crohn's Disease. Am J Gastroenterol.

[19] Seo H, Lee S, So H (2017). Temporal trends in the misdiagnosis rates between Crohn's disease and intestinal tuberculosis. World J Gastroenterol.

[20] Patton PH, Parker CE, MacDonald JK, Chande N. Anti-tuberculous therapy for maintenance of remission in Crohn's disease. Cochrane Database Syst Rev. 2016;7(7):CD000299. 10.1002/14651858.CD000299.pub3 PMC6457855 27444319

[21] Pratap Mouli V, Munot K, Ananthakrishnan A (2017). Endoscopic and clinical responses to anti-tubercular therapy can differentiate intestinal tuberculosis from Crohn's disease. Aliment Pharmacol Ther.

[22] Sharma V, Mandavdhare HS, Lamoria S, Singh H, Kumar A (2018). Serial C-reactive protein measurements in patients treated for suspected abdominal tuberculosis. Dig Liver Dis.

[23] Sharma V, Verma S, Kumar-M P (2021). Serial measurements of faecal calprotectin may discriminate intestinal tuberculosis and Crohn's disease in patients started on antitubercular therapy. Eur J Gastroenterol Hepatol.

[24] Gupta A, Pratap Mouli V, Mohta S (2020). Antitubercular Therapy Given to Differentiate Crohn's Disease From Intestinal Tuberculosis Predisposes to Stricture Formation. J Crohns Colitis.

[25] Banerjee R, Pal P, Girish BG, Reddy DN (2018). Risk factors for diagnostic delay in Crohn's disease and their impact on long-term complications: how do they differ in a tuberculosis endemic region?. Aliment Pharmacol Ther.

[26] Liu F, Tang J, Ye L (2022). Prophylactic Antitubercular Therapy Is Associated With Accelerated Disease Progression in Patients With Crohn's Disease Receiving Anti-TNF Therapy: A Retrospective Multicenter Study. Clin Transl Gastroenterol.

[27] Jena A, Jha DK, Sharma V (2021). Distinguishing intestinal tuberculosis from Crohn's disease. Lancet Gastroenterol Hepatol.

[28] Fehily SR, Al-Ani AH, Abdelmalak J (2022). Review article: latent tuberculosis in patients with inflammatory bowel diseases receiving immunosuppression-risks, screening, diagnosis and management. Aliment Pharmacol Ther.

[29] Kolhe K (2022). Sharma V : Differentiating Gastrointestinal Tuberculosis and Crohn’s Disease—Antitubercular Therapy. Corticosteroids or Both: J Gastrointest Infect.

[30] Panigrahi MK, Kumar C (2022). Use of Steroids in Diagnostic Confusion between Intestinal Tuberculosis and Crohn’s Disease: A Brief Experience: J Gastrointest Infect.

[31] Sato R, Nagai H, Matsui H (2019). Ten Cases of Intestinal Tuberculosis Which Were Initially Misdiagnosed as Inflammatory Bowel Disease. Intern Med.

[32] Makharia GK, Srivastava S, Das P (2010). Clinical, endoscopic, and histological differentiations between Crohn's disease and intestinal tuberculosis. Am J Gastroenterol.

[33] Kedia S, Sharma R, Sreenivas V (2017). Accuracy of computed tomographic features in differentiating intestinal tuberculosis from Crohn's disease: a systematic review with meta-analysis. Intest Res.

[34] Sharma V, Singh H, Mandavdhare HS (2017). Tubercular Abdominal Cocoon: Systematic Review of an Uncommon Form of Tuberculosis. Surg Infect (Larchmt).

[35] Cheng L, Huang MF, Mei PF, Bo WH, Deng CS (2013). Zhonghua Nei Ke Za Zhi.

[36] Almadi MA, Ghosh S, Aljebreen AM (2009). Differentiating intestinal tuberculosis from Crohn's disease: a diagnostic challenge. Am J Gastroenterol.

[37] Marshall JB (1993). Tuberculosis of the gastrointestinal tract and peritoneum. Am J Gastroenterol.

[38] Mandavdhare HS, Singh H, Dutta U, Sharma V (2019). A real-world experience with 6 months of antitubercular therapy in abdominal tuberculosis. JGH Open.

[39] Epstein D, Watermeyer G, Kirsch R (2007). Review article: the diagnosis and management of Crohn's disease in populations with high-risk rates for tuberculosis. Aliment Pharmacol Ther.

[40] Amarapurkar DN, Patel ND, Rane PS (2008). Diagnosis of Crohn's disease in India where tuberculosis is widely prevalent. World J Gastroenterol.

[41] Chen W, Fan JH, Luo W, Peng P, Su SB (2013). Effectiveness of interferon-gamma release assays for differentiating intestinal tuberculosis from Crohn's disease: a meta-analysis. World J Gastroenterol.

[42] Mitsuyama K, Niwa M, Takedatsu H (2016). Antibody markers in the diagnosis of inflammatory bowel disease. World J Gastroenterol.

[43] Ng SC, Hirai HW, Tsoi KK (2014). Systematic review with meta-analysis: accuracy of interferon-gamma releasing assay and anti-Saccharomyces cerevisiae antibody in differentiating intestinal tuberculosis from Crohn's disease in Asians. J Gastroenterol Hepatol.

[44] Makharia GK, Sachdev V, Gupta R, Lal S, Pandey RM (2007). Anti-Saccharomyces cerevisiae antibody does not differentiate between Crohn's disease and intestinal tuberculosis. Dig Dis Sci.

[45] Zhang S, Luo J, Wu Z (2018). Antibodies against glycoprotein 2 display diagnostic advantages over ASCA in distinguishing CD from intestinal tuberculosis and intestinal Behçet's disease. Clin Transl Gastroenterol.

[46] Jiang M, Zeng Z, Chen K (2022). Enterogenous Microbiotic Markers in the Differential Diagnosis of Crohn's Disease and Intestinal Tuberculosis. Front Immunol.

[47] Ghoshal UC, Ghoshal U, Singh H, Tiwari S (2007). Anti-Saccharomyces cerevisiae antibody is not useful to differentiate between Crohn's disease and intestinal tuberculosis in India. J Postgrad Med.

[48] Dutta AK, Sahu MK, Gangadharan SK, Chacko A (2011). Distinguishing Crohn's disease from intestinal tuberculosis–a prospective study. Trop Gastroenterol.

[49] Shah S, Thomas V, Mathan M (1992). Colonoscopic study of 50 patients with colonic tuberculosis. Gut.

[50] Kim YG, Kim KJ, Min YK (2020). Comparison of small bowel findings using capsule endoscopy between Crohn's disease and intestinal tuberculosis in Korea. Yeungnam Univ J Med.

[51] Lee YJ, Yang SK, Byeon JS (2006). Analysis of colonoscopic findings in the differential diagnosis between intestinal tuberculosis and Crohn's disease. Endoscopy.

[52] Adult abdominal tuberculosis. Standard Treatment Workflows of India, 2022, Special Edition on Paediatric and Extrapulmonary Tuberculosis, Indian Council of Medical Research, Department of Health Research, Ministry of Health and Family Welfare, Government of India Accessed https://stw.icmr.org.in/images/Adult_Extr_Tuberculosis/1_Adult_Abdominal_TB_18032022.pdf on 1st February 2023.

[53] Kedia S, Ahuja V. Intestinal tuberculosis: an overview. In: Sharma V, ed. Tuberculosis of the Gastrointestinal System. Singapore: Springer; 2022. 10.1007/978-981-16-9053-2_6.

[54] Kapoor VK, Chattopadhyay TK, Sharma LK (1988). Radiology of abdominal tuberculosis. Australas Radiol.

[55] Lee SS, Kim AY, Yang SK (2009). Crohn disease of the small bowel: comparison of CT enterography, MR enterography, and small-bowel follow-through as diagnostic techniques. Radiology.

[56] Goodsall TM, Jairath V, Feagan BG (2021). Standardisation of intestinal ultrasound scoring in clinical trials for luminal Crohn's disease. Aliment Pharmacol Ther.

[57] Goyal P, Shah J, Gupta S, Gupta P, Sharma V (2019). Imaging in discriminating intestinal tuberculosis and Crohn's disease: past, present and the future. Expert Rev Gastroenterol Hepatol.

[58] Kalra N, Agrawal P, Mittal V (2014). Spectrum of imaging findings on MDCT enterography in patients with small bowel tuberculosis. Clin Radiol.

[59] Paulsen SR, Huprich JE, Fletcher JG (2006). CT enterography as a diagnostic tool in evaluating small bowel disorders: review of clinical experience with over 700 cases. Radiographics.

[60] Smith EA, Dillman JR, Adler J, Dematos-Maillard VL, Strouse PJ (2012). MR enterography of extraluminal manifestations of inflammatory bowel disease in children and adolescents: moving beyond the bowel wall. AJR Am J Roentgenol.

[61] Koh DM, Miao Y, Chinn RJ (2001). MR imaging evaluation of the activity of Crohn's disease. AJR Am J Roentgenol.

[62] Seth R, Gupta P, Debi U (2023). Perfusion computed tomography may help in discriminating gastrointestinal tuberculosis and Crohn’s disease : Diagnostics.

[63] Ilvemark JFKF, Hansen T, Goodsall TM (2022). Defining Transabdominal Intestinal Ultrasound Treatment Response and Remission in Inflammatory Bowel Disease: Systematic Review and Expert Consensus Statement. J Crohns Colitis.

[64] Ma L, Li W, Zhuang N (2021). Comparison of transmural healing and mucosal healing as predictors of positive long-term outcomes in Crohn's disease. Therap Adv Gastroenterol.

[65] Ma L, Zhu Q, Li Y (2019). The potential role of CT enterography and gastrointestinal ultrasound in the evaluation of anti-tubercular therapy response of intestinal tuberculosis: a retrospective study. BMC Gastroenterol.

[66] Mathur P, Sharma R, Kandasamy D, Kedia S, Gamanagatti S, Ahuja V (2019). Can ADC be used as a surrogate marker of response to therapy in intestinal tuberculosis?. Abdom Radiol (NY).

[67] Makanjuola D (1998). Is it Crohn's disease or intestinal tuberculosis?. CT analysis Eur J Radiol.

[68] Pulimood AB, Amarapurkar DN, Ghoshal U (2011). Differentiation of Crohn's disease from intestinal tuberculosis in India in 2010. World J Gastroenterol.

[69] Ye Z, Lin Y, Cao Q, He Y, Xue L (2015). Granulomas as the Most Useful Histopathological Feature in Distinguishing between Crohn's Disease and Intestinal Tuberculosis in Endoscopic Biopsy Specimens. Medicine (Baltimore).

[70] Tandon HD, Prakash A (1972). Pathology of intestinal tuberculosis and its distinction from Crohn's disease. Gut.

[71] Price AB, Morson BC (1975). Inflammatory bowel disease: the surgical pathology of Crohn's disease and ulcerative colitis. Hum Pathol.

[72] Lockhart-Mummery HE, Morson BC (1964). Crohn's disease of the large intestine. Gut.

[73] Pulimood AB, Ramakrishna BS, Kurian G (1999). Endoscopic mucosal biopsies are useful in distinguishing granulomatous colitis due to Crohn's disease from tuberculosis. Gut.

[74] Du J, Ma YY, Xiang H, Li YM (2014). Confluent granulomas and ulcers lined by epithelioid histiocytes: new ideal method for differentiation of ITB and CD? A meta analysis. PLoS ONE.

[75] Watermeyer GA, Locketz M (2018). CD73 expression in tissue granulomas in distinguishing intestinal tuberculosis from Crohn's disease in a South African cohort. Scand J Gastroenterol.

[76] Mehta V, Desai D, Abraham P (2018). Do additional colonoscopic biopsies increase the yield of Mycobacterium tuberculosis culture in suspected ileo-colonic tuberculosis?. Indian J Gastroenterol.

[77] Jin T, Fei B, Zhang Y, He X (2017). The diagnostic value of polymerase chain reaction for *Mycobacterium tuberculosis* to distinguish intestinal tuberculosis from crohn's disease: A meta-analysis. Saudi J Gastroenterol.

[78] Sharma V, Soni H, Kumar-M P (2021). Diagnostic accuracy of the Xpert MTB/RIF assay for abdominal tuberculosis: a systematic review and meta-analysis. Expert Rev Anti Infect Ther.

[79] Sharma K, Sharma M, Sharma V (2022). Evaluating diagnostic performance of Truenat MTB Plus for gastrointestinal tuberculosis. J Gastroenterol Hepatol.

[80] Hallur V, Sharma M, Sethi S (2013). Development and evaluation of multiplex PCR in rapid diagnosis of abdominal tuberculosis. Diagn Microbiol Infect Dis.

[81] Li X, Liu X, Zou Y, et al. Predictors of clinical and endoscopic findings in differentiating Crohn's disease from intestinal tuberculosis [published correction appears in Dig Dis Sci. 2011 Mar;56(3):920]. Dig Dis Sci. 2011;56(1):188–196. 10.1007/s10620-010-1231-4 20467901

[82] Yu H, Liu Y, Wang Y, Peng L, Li A, Zhang Y (2012). Clinical, endoscopic and histological differentiations between Crohn's disease and intestinal tuberculosis. Digestion.

[83] Jung Y, Hwangbo Y, Yoon SM (2016). Predictive factors for differentiating between Crohn's disease and intestinal tuberculosis in Koreans. Am J Gastroenterol.

[84] Mao R, Liao WD, He Y (2015). Computed tomographic enterography adds value to colonoscopy in differentiating Crohn's disease from intestinal tuberculosis: a potential diagnostic algorithm. Endoscopy.

[85] Park MJ, Lim JS (2013). Computed tomography enterography for evaluation of inflammatory bowel disease. Clin Endosc.

[86] Zhao XS, Wang ZT, Wu ZY (2014). Differentiation of Crohn's disease from intestinal tuberculosis by clinical and CT enterographic models. Inflamm Bowel Dis.

[87] Zhang T, Fan R, Wang Z (2015). Differential diagnosis between Crohn's disease and intestinal tuberculosis using integrated parameters including clinical manifestations, T-SPOT, endoscopy and CT enterography. Int J Clin Exp Med.

[88] Kedia S, Sharma R, Nagi B (2015). Computerized tomography-based predictive model for differentiation of Crohn's disease from intestinal tuberculosis. Indian J Gastroenterol.

[89] Huang X, Liao WD, Yu C (2015). Differences in clinical features of Crohn's disease and intestinal tuberculosis. World J Gastroenterol.

[90] Bae JH, Park SH, Ye BD (2017). Development and validation of a novel prediction model for differential diagnosis between Crohn's disease and intestinal tuberculosis. Inflamm Bowel Dis.

[91] Wu X, Huang H, Hou H (2018). Diagnostic Performance of a 5-Marker Predictive Model for Differential Diagnosis Between Intestinal Tuberculosis and Crohn's Disease. Inflamm Bowel Dis.

[92] He Y, Zhu Z, Chen Y (2019). Development and Validation of a Novel Diagnostic Nomogram to Differentiate Between Intestinal Tuberculosis and Crohn's Disease: A 6-year Prospective Multicenter Study. Am J Gastroenterol.

[93] Limsrivilai J, Lee CK, Prueksapanich P (2020). Validation of models using basic parameters to differentiate intestinal tuberculosis from Crohn's disease: A multicenter study from Asia. PLoS ONE.

[94] Yin Y, Zhu ZX, Li Z, Chen YS, Zhu WM (2021). Role of mesenteric component in Crohn's disease: A friend or foe?. World J Gastrointest Surg.

[95] Ko JK, Lee HL, Kim JO (2014). Visceral fat as a useful parameter in the differential diagnosis of Crohn's disease and intestinal tuberculosis. Intest Res.

[96] Yadav DP, Madhusudhan KS, Kedia S (2017). Development and validation of visceral fat quantification as a surrogate marker for differentiation of Crohn's disease and intestinal tuberculosis. J Gastroenterol Hepatol.

[97] Kedia S, Madhusudhan KS, Sharma R (2018). Combination of increased visceral fat and long segment involvement: Development and validation of an updated imaging marker for differentiating Crohn's disease from intestinal tuberculosis. J Gastroenterol Hepatol.

[98] Tiwari V, Kedia S, Garg SK (2018). CD4+ CD25+ FOXP3+ T cell frequency in the peripheral blood is a biomarker that distinguishes intestinal tuberculosis from Crohn's disease. PLoS ONE.

[99] Gupta A, Sharma K, Sharma V (2022). Comparative evaluation of interleukin-10, transforming growth factor-β, and interleukin-17 in gastrointestinal tuberculosis and crohn's disease. Int J Mycobacteriol.

[100] Das P, Rampal R, Udinia S (2018). Selective M1 macrophage polarization in granuloma-positive and granuloma-negative Crohn's disease, in comparison to intestinal tuberculosis. Intest Res.

[101] Zhang F, Xu C, Ning L, et al. Exploration of serum proteomic profiling and diagnostic model that differentiate Crohn's disease and intestinal tuberculosis [published correction appears in PLoS One. 2019 Feb 7;14(2):e0212300]. PLoS One. 2016;11(12):e0167109. 10.1371/journal.pone.0167109 PMC5173341 27997555

[102] Ning L, Shan G, Sun Z (2019). Serum proteome profiles to differentiate Crohn disease from intestinal tuberculosis and primary intestinal lymphoma: A pilot study. Medicine (Baltimore).

[103] Rukmangadachar LA, Makharia GK, Mishra A (2016). Proteome analysis of the macroscopically affected colonic mucosa of Crohn's disease and intestinal tuberculosis. Sci Rep.

[104] Mao H, Su P, Qiu W, Huang L, Yu H, Wang Y (2016). The use of Masson's trichrome staining, second harmonic imaging and two-photon excited fluorescence of collagen in distinguishing intestinal tuberculosis from Crohn's disease. Colorectal Dis.

[105] Tong Y, Lu K, Yang Y (2020). Can natural language processing help differentiate inflammatory intestinal diseases in China? Models applying random forest and convolutional neural network approaches. BMC Med Inform Decis Mak.

[106] Kim JM, Kang JG, Kim S, Cheon JH (2021). Deep-learning system for real-time differentiation between Crohn's disease, intestinal Behçet's disease, and intestinal tuberculosis. J Gastroenterol Hepatol.

[107] Zhu C, Yu Y, Wang S (2021). A Novel Clinical Radiomics Nomogram to Identify Crohn's Disease from Intestinal Tuberculosis. J Inflamm Res.

[108] Lu Y, Chen Y, Peng X (2021). Development and validation of a new algorithm model for differential diagnosis between Crohn's disease and intestinal tuberculosis: a combination of laboratory, imaging and endoscopic characteristics. BMC Gastroenterol.

[109] Logan VS (1969). Anorectal tuberculosis. Proc R Soc Med.

[110] Jena A, Mohindra R, Rana K (2023). Frequency, outcomes, and need for intervention in stricturing gastrointestinal tuberculosis: a systematic review and meta-analysis. BMC Gastroenterol.

[111] Sharma V, Mandavdhare HS, Dutta U (2018). Letter: mucosal response in discriminating intestinal tuberculosis from Crohn's disease-when to look for it?. Aliment Pharmacol Ther.

[112] Lin PY, Wang JY, Hsueh PR (2009). Lower gastrointestinal tract tuberculosis: an important but neglected disease. Int J Colorectal Dis.

[113] Ooi CJ, Makharia GK, Hilmi I, et al. Asia Pacific Consensus Statements on Crohn's disease. Part 1: Definition, diagnosis, and epidemiology: (Asia Pacific Crohn's Disease Consensus--Part 1). J Gastroenterol Hepatol. 2016;31(1):45–55. 10.1111/jgh.12956 25819140

[114] Ran Z, Wu K, Matsuoka K (2021). Asian organization for Crohn's and Colitis and Asia pacific association of gastroenterology practice recommendations for medical management and monitoring of inflammatory bowel disease in Asia. J Gastroenterol Hepatol.

[115] Tahiri M, Goh KL, Abbas Z, Epstein D, Min-Hu C, Mulder CJJ (2023). Digestive tract tuberculosis guideline. J Clin Gastroenterol.

